# Transcatheter edge-to-edge-repair of functional mitral regurgitation induces significant remodeling of mitral annular geometry

**DOI:** 10.3389/fcvm.2023.1143702

**Published:** 2023-06-23

**Authors:** Michael Paukovitsch, Dominik Felbel, Madeleine Jandek, Mirjam Keßler, Wolfgang Rottbauer, Sinisa Markovic, Matthias Groeger, Marijana Tadic, Leonhard Moritz Schneider

**Affiliations:** Department of Cardiology, University Heart Center Ulm, Ulm, Germany

**Keywords:** functional mitral regurgitation (FMR), anterior-posterior mitral annulus diameter, transcatheter edge-to-edge repair, transesophageal echocardiography, annuloplasty

## Abstract

**Background:**

Mitral annular alterations in the context of heart failure often lead to severe functional mitral regurgitation (FMR), which should be treated with transcatheter edge-to-edge repair (M-TEER) according to current guidelines. M-TEER's effects on mitral valve (MV) annular remodeling have not been well elucidated.

**Methods:**

141 consecutive patients undergoing M-TEER for treatment of FMR were included in this investigation. Comprehensive intraprocedural transesophageal echocardiography was used to assess the acute effects of M-TEER on annular geometry.

**Results:**

Average patient age was 76.2 ± 9.6 years and 46.1% were female patients. LV ejection fraction was reduced (37.0% ± 13.7%) and all patients had mitral regurgitation (MR) grade ≥III. M-TEER achieved optimal MR reduction (MR ≤ I) in 78.6% of patients. Mitral annular anterior-posterior diameters (A-Pd) were reduced by −6.2% ± 9.5% on average, whereas anterolateral-posteromedial diameters increased (3.7% ± 8.9%). Overall, a reduction in MV annular areas was observed (2D: −1.8% ± 13.1%; 3D: −2.7% ± 13.7%), which strongly correlated with A-Pd reduction (2D: *r* = 0.6, *p* < 0.01; 3D: *r* = 0.65, *p* < 0.01). Patients that achieved A-Pd reduction above the median (≥6.3%) showed significantly lower rates of the composite endpoint rehospitalization for heart failure or all-cause mortality than those with less A-Pd reduction (9.9% vs. 28.6%, *p* = 0.037, log-rank *p* = 0.039). Furthermore, patients reaching the composite endpoint had an increase in annular area (2D: 3.0% ± 15.4%; 3D: 1.9% ± 15.3%), whereas those not reaching the endpoint showed a decrease (2D: −2.7% ± 12.4%; 3D: −3.6% ± 13.3%), although residual MR after M-TEER was similar between these groups (*p* = 0.57). In multivariate Cox regression adjusted for baseline MR, A-Pd reduction ≥6.3% remained a significant predictor of the combined endpoint (OR: 0.35, 95% CI: 0.14–0.85, *p* = 0.02).

**Conclusion:**

Our findings indicate that effects of M-TEER in FMR are not limited to MR reduction, but also have significant impact on annular geometry. Moreover, A-Pd reduction, which mediates annular remodeling, has a significant impact on clinical outcome independent of residual MR.

## GRAPHICAL ABSTRACT

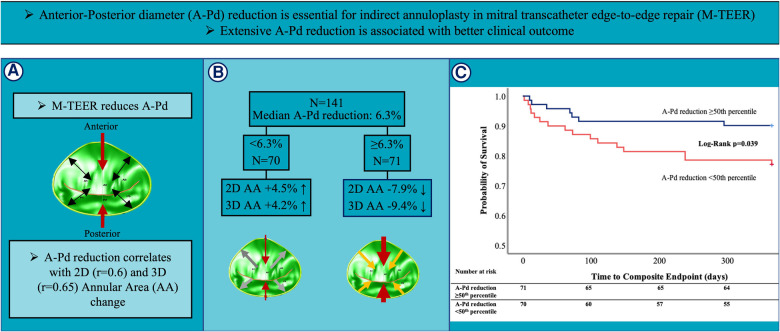

Summary of study design and key results of this comprehensive 3D TEE analysis investigating alterations in MV annular geometry during M-TEER and its impact on outcome in 141 FMR patients. (A) M-TEER reduces A-Pd (red arrows) and induces changes in annular geometry (black arrows). (B) 3D TEE assessment showed a median A-Pd reduction of 6.3%. In patients with less extensive A-Pd reduction (<6.3%) 2D and 3D AA increased (white arrows), whereas patients with extensive A-Pd reduction (≥6.3%) showed 2D and 3D AA decrease (orange arrows). (C) Kaplan-Meier analysis for the composite endpoint of rehospitalization for heart failure and all-cause mortality showed significantly better outcomes in patients with extensive A-Pd reduction.

## Introduction

Mitral transcatheter edge-to-edge repair (M-TEER) is both an established and foremost minimally invasive treatment for symptomatic degenerative (DMR) and functional (FMR) mitral regurgitation. In particular, FMR has become the dominating etiology in patients treated with M-TEER (1–3) due to a lack of other treatment options and its acceptable interventional risk (4, 5). A randomized-controlled trial was able to prove lower rates of rehospitalization for heart failure as well as lower all-cause mortality after M-TEER compared to optimal medical therapy in selected patients with FMR (4). Accordingly, this is reflected in a higher level of recommendation for FMR compared to DMR in current guidelines (6, 7). Using a single or multiple devices, M-TEER alters mitral annular geometry (8–11) and reduces annular anterior-posterior diameters (A-Pd) (8–10, 12, 13). Based on few available studies, these changes are more pronounced in FMR compared to DMR (14) or lack entirely in DMR (8). Furthermore, A-Pd reduction has been suggested to correlate with improved symptomatic patient outcome (8, 12, 14, 15) especially in FMR patients (8). Unlike DMR, FMR is not a pure valvular disease, but rather the consequence of atrial or ventricular impairment affecting the mitral valve (MV) apparatus.

We hypothesized that A-Pd reduction might be an important underlying mechanism in countering the cause of disease in FMR patients and could also correspond to favorable outcomes. In order to investigate the impact and effects of A-Pd reduction, we analyzed FMR patients undergoing M-TEER according to the extent of AP-d reduction.

## Methods

### Study population

This is a retrospective, single-center study that included 141 consecutive FMR patients undergoing M-TEER between October 2019 and September 2021 at the University Hospital Ulm. 149 (64.5%) out of 231 patients treated with M-TEER during the enrollment period suffered from FMR. 2 FMR patients undergoing reintervention were excluded and 6 FMR patients were found to have insufficient image quality for proper 3D analysis. Eventually, subgroups of classical ventricular and atrial FMR were differentiated. Patients with preserved LV ejection fraction and left atrial dilation as the main mechanism of MR were classified as having isolated atrial FMR, whereas patients showing impaired LV function and significant leaflet tethering were classified as having ventricular FMR.

We investigated the acute changes in MV annular geometry during M-TEER procedures performed with the two commercially available M-TEER systems (MitraClip™ Abbott Vascular, Santa Clara, CA, USA and PASCAL™, Edwards Lifesciences, Irvine, CA, USA). All included patients had symptomatic moderate-to-severe (III) or severe (IV) FMR, which remained symptomatic despite guideline-directed medical therapy. All patients were evaluated by the local heart team and referred to M-TEER.

Written informed consent was obtained from all patients prior to data collection. This study was approved by the local ethics board and complies with the Declaration of Helsinki. The authors declare that all supporting data are available within the article and its Online Supplementary Files.

### M-TEER procedure and echocardiography

All procedures were performed by our local team of interventional cardiologists specialized in M-TEER. A treatment strategy was set out by the interventionalists in concurrence with the interventional imagers for each individual patient. Transesophageal echocardiography (TEE) and fluoroscopy were used for procedural guidance. Details of M-TEER have been described elsewhere (16). M-TEER was performed under general anesthesia using either the MitraClip™ third and fourth generation (NTR, XTR, NT, NTW, XT, XTW) or the PASCAL™ first and second generation (P10, Ace) repair systems. Choice of type and number of devices to be implanted were based on a combination of factors including MR jet width and location, MV leaflet length and MV orifice area. In patients with A-Pd ≥36 mm and leaflet lengths ≥9 mm rather large devices, whereas in pathologies showing broad MR jets wider devices were implanted.

2D and 3D MV imaging were employed for device positioning and leaflet grasping. MV gradients and orifice areas were measured before device positioning as well as before and after device deployment. MR was assessed based on an integrative approach with qualitative and quantitative parameters according to current guidelines (17, 18).

In preprocedural transthoracic echocardiography, standard views (apical 4/3/2-chamber, parasternal long-axis and short-axis, subcostal views) were obtained for evaluation of heart chambers and function (see Table 1). Left ventricular (LV) ejection fraction and volumes were calculated using the Simpson's biplane method. Philips EPIQ™ ultrasound system and the X8-2t probe were used for TEE and the X5-1 probe for transthoracic echocardiographic examinations.

**Table 1:** Baseline patient characteristics and echocardiography.

	Total (*N* = 141)	A-Pd reduction ≥6.3% (*N* = 71)	A-Pd reduction <6.3% (*N* = 70)	*p*-Value
Age (years)	76.2 ± 9.6	75.5 ± 9.7	77.0 ± 9.6	0.34
BMI (kg/m^2^)	26.0 ± 5.3	25.7 ± 5.7	26.2 ± 4.9	0.57
Female, *N* (%)	65 (46.1)	36 (50.7)	29 (41.4)	0.31
Arterial hypertension, *N* (%)	109 (77.3)	57 (80.3)	52 (74.3)	0.43
CAD, *N* (%)	90 (63.8)	43 (60.6)	47 (67.1)	0.48
Prior MI	43 (30.5)	22 (31.0)	21 (30.0)	1.0
Hyperlipidemia, *N* (%)	88 (56.3)	40 (56.3)	48 (68.6)	0.17
Pulmonary hypertension, *N* (%)	52 (36.9)	24 (33.8)	28 (40.0)	0.49
COPD, *N* (%)	14 (9.9)	10 (14.1)	4 (5.7)	0.16
Family disposition, *N* (%)	19 (13.6)	8 (11.4)	11 (15.7)	0.62
AFib, *N* (%)	98 (69.5)	46 (64.8)	52 (74.3)	0.27
CRT-D/P, *N* (%)	15 (10.6)	7 (9.9)	8 (11.4)	0.79
DCM, *N* (%)	37 (26.2)	17 (23.9)	20 (28.6)	0.57
NYHA II, *N* (%)	15 (10.6)	9 (12.7)	6 (8.6)	0.67
NYHA III, *N* (%)	100 (70.9)	50 (70.4)	50 (71.4)	
NYHA IV, *N* (%)	26 (18.4)	12 (16.9)	14 (20.0)	
Euro SCORE II	6.3 ± 6.1	5.2 ± 7.2	6.5 ± 4.8	0.34
STS score	4.9 ± 5.0	4.4 ± 5.3	5.5 ± 4.6	0.2
Troponin T pre (µg/L)	30.0 (18.0–45.0)	29.5 (21.0–45.0)	31.0 (17.0–47.0)	0.94
NT-proBNP pre (pg/ml)	2,953 (1,323–5,969)	3,109 (1,396.5–6,750.5)	2,546 (1,189.0–5,307.0)	0.23
eGFR (ml/min)	46.6 ± 19.1	46.2 ± 19.3	47.03 ± 19.1	0.8
CKD III/IV	107 (76.4)	55 (77.5)	52 (75.4)	0.84
BB, *N* (%)	119 (84.4)	62 (87.3)	57 (81.4)	0.36
ACEI, *N* (%)	34 (24.1)	15 (21.1)	19 (27.1)	0.44
ARB, *N* (%)	43 (30.5)	21 (29.6)	22 (31.4)	0.86
ARNI, *N* (%)	41 (29.1)	19 (26.8)	22 (31.4)	0.58
MRA, *N* (%)	86 (61.0)	40 (45.3)	46 (65.7)	0.3
SGLT-2 inhibitors, *N* (%)	19 (13.5)	5 (7.0)	14 (20.0)	0.03
Loop diuretics, *N* (%)	118 (83.7)	58 (81.7)	60 (85.7)	0.65
Statins, *N* (%)	97 (68.8)	49 (69.0)	48 (68.6)	1.0
ASS, *N* (%)	37 (26.2)	22 (31.0)	15 (21.4)	0.25
NOAC, *N* (%)	87 (61.7)	39 (54.9)	48 (68.6)	0.12
P2Y12 inhibitor, *N* (%)	42 (29.8)	24 (33.8)	18 (25.7)	0.36
LVEF (%)	37.0 ± 13.7	38.0 ± 13.8	36.0 ± 13.7	0.43
LVEDd (mm)	61.0 ± 13.2	60.5 ± 12.6	61.5 ± 14.0	0.69
LVEDV (mm)	174.1 ± 83.5	176.2 ± 91.7	171.9 ± 74.4	0.78
LVESd (mm)	48.0 ± 14.7	47.4 ± 13.3	48.6 ± 16.4	0.69
LVESV (ml)	109.2 ± 73.4	110.7 ± 80.4	107.6 ± 66.5	0.82
LA Diameter (mm)	55.3 ± 10.8	55.2 ± 12.6	55.3 ± 8.6	0.98
TAPSE (mm)	17.9 ± 4.6	17.9 ± 4.5	17.9 ± 4.8	0.97
sPAP (mmHg)	51.1 ± 16.4	51.3 ± 16.0	50.8 ± 16.9	0.88
Average grade of TR	1.9 ± 0.9	1.8 ± 1.0	2.0 ± 0.9	0.32
Severe TR, *N* (%)	41 (29.1)	19 (26.8)	22 (31.4)	0.54
Average Grade of MR pre	3.6 ± 0.5	3.7 ± 0.5	3.5 ± 0.5	**0.02**
MR grade III, *N* (%)	57 (40.4)	22 (31.0)	35 (50.0)	**0.02**
MR grade IV, *N* (%)	84 (59.6)	49 (69.0)	35 (50.0)	
Mean PG (mmHg) pre	2.3 ± 1.5	2.0 ± 1.0	2.7 ± 1.7	**<0.01**
ERO A (cm^2^)	0.3 ± 0.1	0.3 ± 0.2	0.3 ± 0.1	0.97
Vena contracta (mm)	8.8 ± 3.0	8.9 ± 3.3	8.6 ± 2.6	0.49
PISA (cm)	0.8 ± 0.2	0.7 ± 0.2	0.7 ± 0.2	0.59
MR RV (ml)	39.2 ± 19.6	39.4 ± 20.8	39.0 ± 18.6	0.92
MV orifice area pre (cm^2^)	4.1 ± 1.5	4.1 ± 1.5	4.0 ± 1.6	0.89

Values are shown as frequencies (N) and percentages (%), mean ± standard deviation (SD).

BMI, body mass index (kg/m^2^); CAD, coronary artery disease; COPD, chronic obstructive pulmonary disease; AF, atrial fibrillation; LBBB, left bundle branch block; CRT, cardiac resynchronization therapy; DCM, dilatative cardiomyopathy; NYHA, New York heart association; STS, society of thoracic surgeons; NT-proBNP, N-terminal pro hormone brain natriuretic peptide; eGFR, estimated glomerular filtration rate; BP, blood pressure; BB, beta blocker; ACEI, angiotensin converting enzyme inhibitor; ARB, AT receptor blocker; ARNI, angiotensin-neprilysin inhibitor; MRA, mineralocorticoid receptor antagonist; SGLT-2, sodium-glucose cotransporter-2; ASS, acetylic salicylic acid; NOAC, novel oral anticoagulant; P2Y12 inhibitor, adenosine diphosphate receptor antagonists; LVEF, left-ventricular ejection fraction; LVEDd, left-ventricular end-diastolic diameter; LVEDV, left-ventricular end-diastolic volume; LVESd, left-ventricular end-systolic diameter; LVESV, left-ventricular end-systolic volume; LA, left atrium; IVSd, septum diameter; TAPSE, tricuspid annular plane systolic excursion; sPAP, systolic pulmonary artery pressure; TR, tricuspid regurgitation; MR, mitral regurgitation; ERO A, effective regurgitant orifice area; PISA, proximal isovelocity surface area; RV, regurgitant volume.

Bold values indicate significant *p*-values.

### Imaging and quantification of the MV apparatus

2D and 3D TEE images obtained during M-TEER procedure were processed offline using a commercially available semi-automatic assessment tool (TOMTEC-ARENA, TOMTEC Imaging Systems, Munich). This tool allows 4D MV modeling and produces measurements of the MV apparatus such as A-Pd, anterolateral-posteromedial diameter (AL-PMd), annular circumference (AC) as well as 2D and 3D annular areas (AA). Application of this tool requires a 3D image of the MV and landmarks to be set within the MV apparatus in two-chamber and three-chamber views using multiple plane reconstruction (MPR). The reference plane was positioned in line with the MV annulus. This enabled placing the landmarks of leaflet insertion at the MV annulus, orientation of the aortic annulus, and coaptation point. Thus, both static and dynamic 4D models of the MV apparatus were generated and all parameters and their numerical values were measured by the software. These models were optimized manually by adjusting annulus and leaflet contours as well as commissural positions in order to provide better accuracy of the 3D model. Adjusting was performed in different MV planes in order to calculate A-Pd and AL-PMd. For measurements after device deployment, the coaptation point (three-chamber view) was set at the intersection of leaflets and device. In case of two devices the MV coaptation point was set within the device closest to the intersection of the A-Pd and AL-PMd. All parameters were measured in the end-systolic phase of the cardiac cycle. To determine changes in mitral annular geometry, measurements were performed before and after device implantation using images from intraprocedural TEE exclusively. Changes in MV annular diameters were measured in 2D using 3D MPR.

### Follow-up

Follow-up data were collected during clinical visits or telephone interviews performed by trained study nurses. All patients had scheduled appointments at our hospital every 3 months and if they would not show up for any reason, they were called and data was collected remotely. Follow-up was available for at least 12 months.

### Statistical methods

Analysis included evaluation of the whole cohort of patients, as well as a comparison between groups with different levels of relative A-Pd reduction. The median of relative A-Pd reduction was used as cut-off. The validity of this cut-off value (sensitivity and specificity) with regard to the composite endpoint was tested in receiver operating characteristic (ROC) curve. The cut-off value with optimal sensitivity and specificity was calculated using Youden's index. Continuous variables were expressed using mean and standard deviation or median and interquartile range. For paired variables mean change and mean relative change were calculated. Distribution of variables was analyzed graphically using histograms and Q–Q plots. Continuous variables were compared using *t*-test if they showed normal distribution or Wilcoxon test where appropriate. In case of paired variables, the paired Student *t*-test or the Wilcoxon test were utilized. Categorical variables are shown as frequencies and percentages and were compared using Chi-square test or Fisher exact test, where appropriate. Univariate and multivariate binary logistic regression were used to analyze parameters related with A-Pd reduction. Correlation analysis was performed using Pearson and Spearman's correlation coefficients where appropriate. Kaplan–Meier analysis and the log-rank test were used for time-to-event comparison of the composite endpoint of all-cause death or rehospitalization. For outcome analysis and as a primary endpoint the composite endpoint of death and/or rehospitalization for heart failure was used.

For variables significantly differing between patient groups (*p* < 0.05) or possibly impacting the combined endpoint (*p* < 0.2) univariate Cox regression was performed. Multivariate Cox regression included all variables that showed potential influence in the univariate regression analysis (*p* < 0.2). To ensure model stability, collinearity was tested using Spearman's and Pearson's correlation coefficients. A *p*-value of <0.05 was considered statistically significant. Statistical analysis was performed using SPSS, IBM Statistics, Versions 28 and 29 software packages.

## Results

### Patient characteristics and annular change in the overall cohort

In the overall cohort, average patient age was 76.2 ± 9.6 years (see Table 1). The majority (59.6%) of patients suffered from severe (IV) FMR (see Table 1). Optimal MR reduction (MR ≤ I) was achieved in 78.7% of patients (see Table 2). Risk of procedural mortality as defined by the Society of Thoracic Surgeons and EUROScoreII was 4.9% ± 5.0% and 6.3% ± 6.1%, respectively. Technical success was achieved in all patients. An average LV ejection fraction of 37.0% ± 13.7% was observed (see Table 1). Regarding the overall cohort, M-TEER reduced A-Pd (−6.2% ± 9.5%, *p* < 0.01) as well as 2D (−1.8% ± 13.1%, *p* < 0.01) and 3D (−2.7% ± 13.7%, *p* = 0.26) MV AA (see Table 3). AL-PMd increased by 3.7% ± 8.9% (*p* < 0.01).

**Table 2:** Procedural outcomes.

	Total (*N* = 141)	A-Pd reduction ≥6.3% (*N* = 71)	A-Pd reduction <6.3% (*N* = 70)	*p*-Value
Average grade of MR post	1.1 ± 0.6	1.1 ± 0.5	1.1 ± 0.6	0.73
MR grade ≤I, *N* (%)	111 (78.7)	58 (81.7)	53 (75.7)	0.36
MR grade II, *N* (%)	30 (21.3)	13 (18.3)	17 (24.3)	
Mean PG (mmHg) post	3.1 ± 1.2	3.0 ± 1.2	3.1 ± 1.2	0.77
MV orifice area post (cm^2^)	2.7 ± 1.5	2.7 ± 1.3	2.8 ± 1.7	0.78
Number of implanted devices	1.4 ± 0.5	1.4 ± 0.5	1.3 ± 0.5	0.33
Device type				
NTR/NT/NTW, *N* (%)	43 (30.5)	24 (33.8)	19 (27.1)	0.17
XTR/XT/XTW, *N* (%)	25 (17.7)	16 (22.5)	9 (12.9)	
PASCAL P10, *N* (%)	33 (23.4)	16 (22.5)	17 (24.3)	
PASCAL Ace, *N* (%)	40 (28.4)	15 (21.1)	25 (35.7)	

Values are shown as frequencies (N) and percentages (%) or mean ± standard deviation (SD).

MR, mitral regurgitation; MV, mitral valve, PG, pressure gradient.

**Table 3:** 4D MV analysis according to the median relative change in A-Pd reduction.

	Total (*N* = 141)	A-Pd reduction ≥6.3% (*N* = 71)	A-Pd reduction <6.3% (*N* = 70)	*p*-Value
A-Pd pre (cm)	4.0 ± 0.5	4.0 ± 0.5	4.0 ± 0.5	0.84
A-Pd post (cm)	3.8 ± 0.6	3.5 ± 0.5	4.00 ± 0.5	**<0**.**01**
mean relative change (%)	−6.2 ± 9.5	−13.0 ± 6.1	0.8 ± 7.1	**<0**.**01**
***p* (pre-post)**	**<0.01**	**<0.01**	**<0.01**	
AL-PMd pre (cm)	4.1 ± 0.5	4.1 ± 0.5	4.1 ± 0.5	0.56
AL-PMd post (cm)	4.3 ± 0.5	4.2 ± 0.5	4.3 ± 0.5	0.18
mean relative change (%)	3.7 ± 8.9	2.6 ± 7.3	4.9 ± 10.2	0.13
***p* (pre-post)**	**<0.01**	**<0.01**	**<0.01**	
Nonplanar angle pre (°)	152.0 ± 11.6	152.7 ± 11.2	151.3 ± 12.1	0.47
Nonplanar angle post (°)	149.7 ± 13.6	150.7 ± 14.8	150.7 ± 12.4	0.4
mean relative change (%)	−1.2 ± 9.5	−1.2 ± 9.5	−1.3 ± 8.8	0.84
***p* (pre-post)**	0.055	0.26	**<0.01**	
AC pre (cm)	13.5 ± 1.4	13.4 ± 1.4	13.5 ± 1.4	0.53
AC post (cm)	13.3 ± 1.5	12.9 ± 1.5	13.8 ± 1.5	**<0**.**01**
mean relative change (%)	−0.9 ± 6.4	−3.8 ± 4.9	2.0 ± 6.4	**<0**.**01**
***p* (pre-post)**	0.061	**<0.01**	**<0.01**	
2D AA pre (cm^2^)	13.0 ± 2.7	12.8 ± 2.7	13.1 ± 2.8	0.56
2D AA post (cm^2^)	12.7 ± 3.0	11.8 ± 2.7	13.6 ± 3.0	**<0**.**01**
mean relative change (%)	−1.8 ± 13.1	−7.9 ± 8.9	4.5 ± 13.7	**<0**.**01**
***p* (pre-post)**	**0.049**	**<0.01**	**<0.01**	
3D AA pre (cm^2^)	13.6 ± 3.1	13.6 ± 3.3	13.6 ± 2.8	0.92
3D AA post (cm^2^)	13.1 ± 3.1	12.2 ± 2.8	14.1 ± 3.0	**<0**.**01**
mean relative change (%)	−2.7 ± 13.7	−9.4 ± 10.3	4.2 ± 13.4	**<0**.**01**
***p* (pre-post)**	0.26	**<0.01**	**<0.01**	
Tenting volume pre (cm^3^)	4.8 ± 2.6	4.5 ± 2.5	5.2 ± 2.7	0.15
Tenting volume post (cm^3^)	4.7 ± 2.6	4.0 ± 2.2	5.3 ± 2.6	**<0**.**01**
mean relative change (%)	2.1 ± 37.2	−8.1 ± 36.9	12.1 ± 34.9	**<0**.**01**
***p* (pre-post)**	0.27	**0.01**	**<0.01**	
Tenting area pre (cm^2^)	2.7 ± 1.2	2.6 ± 1.2	2.8 ± 1.3	0.3
Tenting area post (cm^2^)	2.4 ± 1.1	2.1 ± 0.9	2.7 ± 1.1	**<0**.**01**
mean relative change (%)	−1.0 ± 58.8	−8.0 ± 71.4	6.0 ± 41.9	0.16
***p* (pre-post)**	**<0.01**	**<0.01**	0.44	
Annular height pre (cm)	1.0 ± 1.3	1.1 ± 1.8	1.0 ± 0.3	0.47
Annular height post (cm)	0.9 ± 0.3	0.8 ± 0.3	0.9 ± 0.3	**<0**.**01**
mean relative change (%)	−4.1 ± 29.3	−9.5 ± 31.6	1.3 ± 25.9	**0**.**03**
***p* (pre-post)**	0.11	0.14	0.16	

AA, Annular area; AC, Annular circumference; AL-PMd, anterolateral-posteromedial diameter; A-Pd, Anterior-posterior diameter; MV, mitral valve.

Values are shown as mean ± standard deviation (SD); *p*(pre-post) refers to testing for paired variables.

Bold numbers indicate significant *p*-values.

For outcome analysis of MV annular change, patients were grouped according to the composite endpoint rehospitalization for heart failure or all-cause mortality one year after M-TEER (see Supplementary Table S1). Follow-up data were available for all included patients. There was no difference in preprocedural A-Pd (4.0 cm ± 0.5 cm vs. 4.1 cm ± 0.6 cm, *p* = 0.22) nor in relative (%) A-Pd change (−6.3 ± 10.1 vs. −5.3% ± 6.5%, *p* = 0.64) between these groups and preprocedural (3.6 ± 0.5 vs. 3.7 ± 0.5, *p* = 0.13) as well as postprocedural (1.1 ± 0.5 vs. 1.2 ± 0.7, *p* = 0.57) MR severity was similar. However, annular size reduction measured as reduction of AA was only observed in patients who did not reach the composite endpoint (2D AA: −2.7% ± 12.4% vs. 3.1% ± 15.4%, *p* = 0.05; 3D AA: −3.6% ± 13.3% vs. 1.9% ± 15.3%, *p* = 0.08). Similarly, paired testing showed that significant annular change occurred only in patients not reaching the composite endpoint [*p*(pre-post) 2D AA: < 0.01 vs. 0.47; 3D: < 0.01 vs. 0.84]. Moreover, further analysis revealed a strong correlation between %A-Pd reduction with %2D and %3D AA reduction (*r* = 0.6, *p* < 0.01; *r* = 0.65, *p* < 0.01; see Table 4). To corroborate these interesting findings, we divided the overall cohort by the median of %A-Pd reduction into one group with extensive and another group showing less extensive A-Pd reduction.

**Table 4:** Correlation analysis for mitral valve annular dimensions.

	Mean % change APd	Mean % change AL-PMd	Mean % change AA 2D	Mean % change AA 3D	Mean % change AC
% Change A-Pd		0.053 (0.54)	0.6 **(<0.01)**	0.65 (**<0.01)**	0.6 (**<0.01)**
% Change AL-PMd			0.74 **(<0.01)**	0.67 (**<0.01)**	0.56 (**<0.01)**
% Change AA 2D				0.97 (**<0.01)**	0.88 (**<0.01)**
% Change AA 3D					0.94 (**<0.01)**
% Change Ac					

Bold numbers indicate significant *p*-values.

### Annular change in patients with extensive A-Pd reduction

Median A-Pd reduction was found to be −6.3% in the overall cohort (interquartile range −1.5% to −12.0%). Accordingly, 71 patients with A-Pd reduction ≥6.3% (extensive) were compared to 70 patients showing <6.3% (less extensive) A-Pd reduction. There were no significant differences regarding baseline characteristics such as age (*p* = 0.34), female gender (*p* = 0.31) or comorbidities like atrial fibrillation (*p* = 0.27) and chronic kidney disease stage III/IV (*p* = 0.84) between both groups (see Table 1). Society of Thoracic Surgeons score (4.4 ± 5.3 vs. 5.5 ± 4.6, *p* = 0.2) and EUROScoreII (5.2 ± 7.2 vs. 6.5 ± 4.8, *p* = 0.34) as well as NYHA class (NYHA III: 70.4% vs. 71.4%, *p* = 0.67) as a surrogate for symptom burden and NT-proBNP (*p* = 0.23) as a biomarker for heart failure were also found to be similar. Except for more frequent use of SGLT2 inhibitors in patients with less extensive A-Pd reduction (7% vs. 20%, *p* = 0.03), there were no significant differences regarding heart failure medication (see Table 1). Preprocedural MR was found to be more severe in patients with extensive A-Pd reduction (MR grade IV: 69.0% vs. 50.0%, *p* = 0.02), while preprocedural mean MV pressure gradients were significantly lower (2.0 ± 1.0 vs. 2.7 ± 1.7 mmHg, *p* < 0.01) in this group (see Table 1). However, no differences were observed regarding postprocedural MR severity (1.1 ± 0.5 vs. 1.1 ± 0.6, *p* = 0.73), optimal MR reduction (residual MR ≤ I: 81.7% vs. 75.4%, *p* = 0.36) and postprocedural mean MV gradients (3.0 ± 1.2 vs. 3.1 ± 1.2 mmHg, *p* = 0.77). Baseline LV end-diastolic volume (176.1 ± 91.7 vs. 171.9 ± 74.4 ml, *p* = 0.78), left atrial diameter (55.2 ± 12.6 vs. 55.3 ± 8.6 mm, *p* = 0.98) and LV ejection fraction were comparable (38.0 ± 13.8 vs. 36.0% ± 13.7%, *p* = 0.43). Single and multiple device implantations were equally prevalent in both groups (single device: 57.7% vs. 65.7%, *p* = 0.39).

Pre- and postprocedural measurements of the MV annulus and corresponding relative changes according to the extent of A-Pd reduction are depicted in Table 3. A histogram of A-Pd change is shown in Supplementary Figure S1. Average relative A-Pd reduction was −13.0% ± 6.1% in patients with extensive compared to 0.8% ± 7.1% in patients with less extensive A-Pd reduction, respectively (*p* < 0.01). At baseline, both groups showed similar A-Pd (4.0 cm ± 0.5 cm vs. 4.0 cm ± 0.5 cm, *p* = 0.84), AL-PMd (4.1 cm ± 0.5 cm vs. 4.1 cm ± 0.5 cm, *p* = 0.56), AC (13.4 cm ± 1.4 cm vs. 13.5 cm ± 1.4 cm, *p* = 0.53) as well as 2D AA (12.8 ± 2.7 vs. 13.1 ± 2.8 cm^2^, *p* = 0.56) and 3D AA (13.6 ± 3.3 vs. 13.6 ± 2.8 cm^2^, *p* = 0.92). After M-TEER, these parameters significantly changed in both groups (see Table 3), however, inverse alterations in annular geometry were observed according to the extent of A-Pd reduction.

Patients with extensive A-Pd reduction showed a decrease in relative change of AC, 2D and 3D AA, whereas these parameters increased in patients with less extensive A-Pd reduction (AC: −3.8% ± 4.9% vs. 2.0% ± 6.4%, *p* < 0.01; 2D AA: −7.9% ± 8.9% vs. 4.5% ± 13.7%, *p* < 0.01; 3D AA: −9.4% ± 10.3% vs. 4.2% ± 13.4%, *p* < 0.01; see also Table 3). Consequently, the decrease in annular sphericity index (ratio of A-Pd/AL-PMd) was more pronounced in patients with extensive A-Pd reduction (−13.8 ± 8.8 vs. −2.4 ± 13.4, *p* < 0.01) and postprocedural comparison of annular dimensions confirms significantly smaller AC (12.9 cm ± 1.5 cm vs. 13.8 cm ± 1.5 cm, *p* < 0.01), 2D AA (11.8 ± 2.7 vs. 13.6 ± 3.0 cm^2^, *p* < 0.01) and 3D AA (12.2 ± 2.8 vs. 14.1 ± 3.0 cm^2^, *p* < 0.01) in this group of patients. AL-PMd increased in both patient groups with a tendency toward greater increase in patients with extensive A-Pd reduction (4.9% ± 10.2% vs. 2.6% ± 7.3%, *p* = 0.13) and the postprocedural annular sphericity index was significantly smaller in these patients (0.94 ± 0.1 vs. 0.83 ± 0.1, *p* < 0.01).

A strong and significant correlation between mean %A-Pd reduction and reduction of mean %AC (*r* = 0.6, *p* < 0.01), %2D AA (*r* = 0.6, *p* < 0.01) and %3D AA (*r* = 0.65, *p* < 0.01) was also found in the study population (see also Table 4). Preprocedural tenting volumes (4.5 ± 2.5 vs. 5.2 ± 2.7 cm^3^, *p* = 0.15) and areas (2.6 ± 1.2 vs. 2.8 ± 1.3 cm^2^, *p* = 0.3) tended to be smaller in patients with extensive A-Pd reduction. This corresponds well with a significantly smaller postprocedural tenting area (2.1 ± 0.9 vs. 2.7 ± 1.1 cm^2^, *p* < 0.01) and volume (4.0 ± 2.2 vs. 5.3 ± 2.6 cm^3^, *p* < 0.01) in these patients. Thus, a significant correlation was also found between %A-Pd reduction and postprocedural tenting area (*r* = 0.4, *p* < 0.01) and volume (*r* = 0.32, *p* < 0.01). Based on the notion that greater preprocedural tenting might explain A-Pd reduction these factors were further tested in logistic regression (see Table 5 and Suplemmental Table 2). Yet neither preprocedural tenting area (OR: 0.86, 95% CI: 0.65–1.14, *p* = 0.3) nor volume (OR: 0.91, 95% CI: 0.8–1.04, *p* = 0.15) were found to be predictors of extensive A-Pd reduction. However, in univariate analysis preprocedural MR severity increased (OR: 2.3, 95% CI: 1.12–4.3, *p* = 0.02), while mean MV pressure gradient decreased the likelihood for extensive A-Pd reduction (OR: 0.64, 95% CI: 0.45–0.91, *p* = 0.01). In multivariate logistic regression, mean MV pressure gradient remained the only significant predictor of extensive A-Pd reduction (OR: 0.65, 95% CI: 0.45–0.93, *p* = 0.02), whereas preprocedural MR severity showed a non-significant tendency (OR: 2.02, 95% CI: 0.94–4.33, *p* = 0.07).

### Outcomes in patients with extensive A-Pd reduction

Outcomes were analyzed using a composite endpoint of rehospitalization and all-cause mortality within the first year after M-TEER. Mean time to follow-up/combined endpoint was 320.8 days (95% CI: 302.6–339.0 days) (Median: 365.0 days, IQR: 365.0–365.0 days). Kaplan–Meier analysis revealed significantly better outcome in patients with greater A-Pd reduction (*p* = 0.039, see Grahical Abstract, Figure 3). The composite endpoint occurred significantly more often in the group with less extensive A-Pd reduction (22.9% vs. 9.9%, *p* = 0.037). Univariate Cox regression analysis (see Table 5 and Supplementary Table S2) demonstrated that preprocedural MR severity (HR: 2.05, 95% CI: 0.81–5.19, *p* = 0.13), mean MV pressure gradient (HR: 1.03, 95% CI: 0.76–1.4, *p* = 0.86) and SGLT-2 inhibitors (HR: 0.57, 95% CI: 0.14–2.45, *p* = 0.45) did not predict the composite endpoint. After adjustment for preprocedural MR severity in multivariate Cox regression, the effect of A-Pd reduction on the composite endpoint remained (HR: 0.35, 95% CI: 0.14–0.85, *p* = 0.02). When testing A-Pd reduction ≥6.3% in multivariate Cox regression together with NT-proBNP and NYHA class, extensive A-Pd reduction also remained a significant predictor of the composite endpoint (HR: 0.31; 95% CI: 0.11–0.89; *p* = 0.03).

**Table 5:** Cox regression for possible predictors of the combined endpoint of death or rehospitalization (further see Supplemental Table S2).

	Univariate			Multivariate		
	HR	95% CI	*p*-Value	HR	95% CI	*p*-Value
NYHA class	2.56	1.19–5.54	**0**.**02**	1.32	0.51	**0**.**57**
NT-proBNP	1.07	1.02–1.13	**0**.**01**	1.08	1.02–1.14	**0**.**01**
A-Pd reduction ≥6.3%	0.41	0.17–0.98	**0**.**046**	0.31	0.11–0.89	**0**.**03**
Mean % change A-Pd	1.01	0.97–1.05	0.63			
A-Pd post	1.6	0.82–3.16	0.17			
Mean % change AC	1.04	0.98–1.11	0.2			
AC post	1.17	0.89–1.52	0.26			
Mean % change AA 2D	1.03	1.002–1.06	**0**.**04**			
AA 2D post	1.12	0.98–1.28	0.1			
Mean % change AA 3D	1.03	0.99–1.06	0.06			
AA 3D post	1.11	0.97–1.26	0.13			
Annular height post	0.4	0.07–2.27	0.3			
Tenting area post	1.18	0.8–1.75	0.41			
Tenting volume post	1.08	0.93–1.27	0.32			
Grade of MR pre	2.05	0.81–5.19	0.13			
mPG pre	1.03	0.76–1.4	0.86			
SGLT-2i	0.57	0.14–2.45	0.45			
A-Pd reduction ≥6.3% (adjusted for grade of preprocedural MR)				0.35	0.14–0.85	**0**.**02**
A-Pd reduction >6.3% (adjusted for grade of preprocedural MR, NT-proBNP and NYHA class)				0.29	0.10–0.83	**0**.**02**

NYHA class, New York Heart association class; NT-proBNP, N-terminal pro hormone brain natriuretic peptide; AA, Annular area; AC, Annular circumference; AL-PMd, anterolateral-posteromedial diameter; A-Pd, anterior-posterior diameter; MR, mitral regurgitation; mPG, mean pressure gradient; SGLT-2i, sodium-glucose cotransporter-2 inhibitor.

Each variable is shown with its odds ratio (OR), respective 95% confidence interval (CI) as well as *p*-value.

Bold numbers indicate significant *p*-values.

To perform a sensitivity analysis regarding the cut-off for relevant A-Pd reduction the coordinates of a receiver operating characteristic (ROC) curve and its respective Youden's index were used. Based upon these calculations, the optimal cut-off value for A-Pd change as a predictor of the composite 1-year endpoint was −6.3% (sensitivity: 0.70, specificity: 0.55).

### Annular change in relevant subgroups

In the overall study cohort, 24 patients were classified as having isolated atrial FMR. The remaining 117 patients showed typical signs of ventricular FMR (see Supplementary Table S3). Both atrial as well as ventricular FMR patients had similar preprocedural AP-d (4.0 cm ± 0.5 cm vs. 4.0 cm ± 0.5 cm, *p* = 0.83) and relative annular change did neither differ regarding A-Pd (*p* = 0.25) nor AL-PM-d (*p* = 0.67), AC (*p* = 0.94) and 2D AA (*p* = 0.75) as well as 3D AA (*p* = 0.68).

In Supplementary Table S4, a comparison of annular geometry and M-TEER induced changes in patients with optimal (MR ≤ I, *N* = 111) and non-optimal (MR ≥ II, *N* = 30) MR results is shown. Patients with optimal MR results showed a non-significant tendency toward greater %AP-d reduction (*p* = 0.2), borderline significance in 2D AA change (*p* = 0.07) and a significant reduction of 3D AA (*p* = 0.02).

Device comparison regarding A-Pd reduction was performed for MitraClip™ vs. PASCAL™ as well as third vs. fourth generation MitraClip™. Significantly greater A-Pd reduction was achieved using the MitraClip™ compared to PASCAL™ (−8.6% ± 9.8% vs. −3.9% ± 8.8%, *p* < 0.01). A comparison of third and fourth generation MitraClip™ did not reveal any relevant differences between devices with or without the option of independent leaflet capture (−8.2% ± 10.1% vs. −8.8% ± 9.8%, *p* = 0.83).

To elucidate the possible influence of a larger spacer as it is a special feature of the original PASCAL™ platform, we also compared A-Pd reduction between the PASCAL™ P10 and other devices (single device procedures). However, no significant differences regarding A-Pd reduction were observed at least in this relatively small group of patients (−5.0% ± 9.0% vs. −6.5% ± 10.5%, *p* = 0.54).

Finally, 18.1% (25/141) of patients in the overall cohort experienced an increase in A-Pd (see also Supplementary Figure S1 and Table S5). These patients also showed a tendency toward greater increase in AL-PMd (3.3% vs. 5.7%, *p* = 0.23) and a significant increase in 2D and 3D AA (2D: 10.0% ± 16.9%, *p* < 0.01; 3D: 10.2% ± 16.8%, *p* < 0.01). Preprocedural annular size and A-Pd did not differ compared to patients with decreasing A-Pd after M-TEER. Notably, mean mitral gradient was significantly greater before (*p* < 0.01) and after device implantation (*p* = 0.02). Additional analysis of anatomical and procedural details in this group of patients revealed frequent commissural device positioning, pronounced and atypical device clocking, incongruity between leaflet and device length as well as a more frequent utilization of shorter MitraClip™ devices (NTR/NT/NTW) and the PASCAL™ Ace.

## Discussion

Our study investigated changes of MV annular geometry during M-TEER and its relationship with 1-year outcomes in FMR patients. It confirmed results of previous studies regarding A-Pd reduction and showed some novel and important findings regarding the acute annular remodeling after M-TEER as well as its impact on outcome of patients with significant FMR. To the best of our knowledge, this is the largest study investigating the impact of A-Pd reduction in FMR patients using comprehensive 3D TEE analysis so far. The main findings of our study can be summarized as follows:

- Extensive A-Pd reduction is associated with significant reductions in AA (2D and 3D), while these parameters increased in patients with less extensive A-Pd reduction.
- Changes in MV geometry, and particularly A-Pd reduction were related with indirect MV annuloplasty.
- M-TEER induced indirect annuloplasty is associated with better clinical outcome represented by a composite endpoint of death or rehospitalization for heart failure.
- Therefore, our study suggests that M-TEER induces changes well beyond leaflet approximation and MR reduction and emphasizes the positive impact of A-Pd reduction on outcome in FMR patients.

The focus of previous studies investigating the effects of M-TEER on MV annular geometry has been directed toward differences in patients with optimal (residual MR ≤ I) and suboptimal/non-optimal (residual MR ≥ II) results (9, 14, 19). Moreover, suboptimal MR reduction was found to be an independent predictor of adverse outcome (14, 19). Obtaining optimal MR reduction is reasonable, however, understanding M-TEER induced changes in annular geometry and their importance for successful treatment go beyond residual MR severity. As shown in our study cohort, extensive A-Pd reduction is associated with favorable outcome independent of residual MR severity. Moreover, comparison of M-TEER induced annular remodeling in patients with optimal and non-optimal MR results emphasizes the importance of indirect annuloplasty in addition to MR reduction.

During M-TEER, one or more devices are usually positioned within the central MV segment between the anterior and posterior leaflet and consequently exert tensile forces on the MV annulus predominantly in anterior-posterior direction. Several studies also using 3D TEE echocardiography were able to show A-Pd reduction during M-TEER (8–12, 14, 15, 20). However, some of these studies did not distinguish between FMR and DMR patients, but provided cumulative results for both entities (9, 13). Other authors selectively included FMR patients (20) or observed A-Pd reduction only among FMR patients in their analyses (8, 11, 12). Few studies reported significant A-Pd reduction in both etiologies (10) with more pronounced A-Pd reduction among FMR patients (15). In terms of additional annular parameters, many investigators similarly reported a decrease in AA (2D or 3D) aside from A-Pd reduction (8, 9, 11, 13, 20), while others did not detect a reduction in AC or AA (14, 20). Based on the number of studies, stronger evidence is found for reduction of AA and AC in FMR (8, 11, 15) compared to DMR (15). Our investigation confirmed M-TEER to reduce A-Pd and extensive A-Pd reduction to be associated with decreased AC and AA. Moreover, we were able to show contrary effects associated with less extensive A-Pd reduction. To our knowledge, no other study investigating M-TEER-induced changes in annular geometry has yet made a similar observation.

The concept of indirect annuloplasty through edge-to-edge repair has already been demonstrated in the earlier days of M-TEER (11). To a certain extent, M-TEER may thus mimic surgical MV repair, where direct annuloplasty through ring implantation is a standard procedure and, interestingly, edge-to-edge repair using the Alfieri stitch was reported to show better outcome when combined with annuloplasty (21, 22). Our study demonstrated M-TEER to be able to induce indirect annuloplasty through A-Pd reduction. However, our findings also suggest that reshaping of the MV annulus requires A-Pd reduction beyond a certain threshold.

Nevertheless, when summarizing the observed changes in our study, no restoration of the saddle-shaped form of the MV annulus occurred. A significant, yet not differing increase in AL-PMd was observed between FMR patients with extensive and less extensive A-Pd reduction. Annular sphericity index significantly decreased when extensive A-Pd reduction occurred. Non-planarity decreased non-significantly in the overall cohort (−1.2% ± 9.5%, *p*{pre-post}=0.055), however, neither preprocedural (*p* = 0.47)/postprocedural (*p* = 0.4) absolute values nor change in non-planarity (*p* = 0.84) differed between patient groups. On the other hand, annular height was reduced significantly greater in patients with extensive A-Pd reduction. Our cohort exclusively consists of FMR patients who typically show some degree of tenting, which is normally reduced after M-TEER (9, 11, 21). In this investigation, tenting was reduced significantly greater in patients with extensive A-Pd reduction (relative tenting volume change: −8.1% ± 36.9% vs. 12.12% ± 34.9%, *p* < 0.01). This seems to come at the expense of saddle-shape restoration as the annulus flattens while being reduced in its overall size (AC, 2D and 3D AA). Given that patients with extensive A-Pd reduction showed greater preprocedural MR severity, yet had similar residual MR after M-TEER compared to those with less A-Pd reduction, it seems as if interventionalists automatically aim at greater A-Pd reduction in the presence of more severe MR. However, this is difficult to prove retrospectively and in multivariate logistic regression for predictors of A-Pd reduction preprocedural MR severity narrowly failed to be a significant predictor (OR: 2.02, 95% CI: 0.94–4.33, *p* = 0.07). MV pressure gradient on the other hand, decreased the likelihood of extensive A-Pd reduction suggesting a possible risk of M-TEER induced MV stenosis (OR: 0.65, 95% CI: 0.45–0.93, *p* = 0.02).

Subgroup analysis of patients with atrial and ventricular FMR showed similar annular size reduction. Tenting was far more pronounced in ventricular FMR, which is inherent to its pathophysiological mechanism. Parameters of annular size and change showed little difference between these etiologies probably due to secondary annular enlargement in ventricular FMR. However, as the number of patients with atrial FMR in our study cohort was small further investigation of differences in atrial and ventricular FMR are warranted.

When investigating device specific differences, the MitraClip™ facilitated significantly greater A-Pd reduction (*p* < 0.01), which could be explained by its stronger mechanical force opposed to the softer and spring-based design of the PASCAL™ platform. Results from a randomized head-to-head comparison between the two M-TEER systems in FMR patients provided by the CLASP IIF trial will further elucidate such differences. No relevant differences were observed between third and fourth generation MitraClip™ (*p* = 0.83) implying that independent leaflet capture is of minor importance particularly in FMR.

The paradoxical increase in A-Pd after M-TEER observed in 25 patients was associated with several anatomical as well as procedural characteristics and their combination. Commissural device positions as well as pronounced and atypical device clocking might induce converse alterations in annular geometry. The use of shorter devices in relation to the respective leaflet length can correct MR. However, it is likely that this mismatch impedes indirect annuloplasty or even causes increase in A-Pd by stretching the MV annulus when LV volume and pressure raises. Finally, utilization of the elastic PASCAL™ design presumably aggravates these conditions.

### Implications of A-Pd reduction for clinical outcome

Few studies have investigated A-Pd reduction in association with patient outcome so far. In a cohort of mixed etiologies, Patzelt et al. observed significantly smaller A-Pd in patients with less residual MR at follow-up and an inverse correlation between these parameters (15). Schueler et al. investigated 111 consecutive patients (71 with FMR) and found acute A-Pd reduction ≥6.4% to significantly predict clinical response (8). In a second cohort, their working group was later able to confirm the finding of favorable clinical outcome in relation to sustained A-Pd reduction ≥6.4% (12). Our study is in concordance with these results. Relative A-Pd reduction above the median of 6.3% was found to significantly predict outcomes after adjusting for preprocedural MR severity in multivariate Cox regression (HR: 0.35, 95% CI: 0.14–0.85, *p* = 0.02) as well as after adjusting in multivariate regression for other outcome related factors such as NT-proBNP, NYHA class and MR severity (HR: 0.31; 95% CI: 0.11–0.89; *p* = 0.03). A-Pd reduction correlated with annular size reduction (AC, 2D and 3D AA) in our study, which implicates that indirect annuloplasty might be responsible for the observed differences in outcome. Eventually, Kreidel et al. demonstrated persistent annular dilation after device implantation in patients with suboptimal results (residual MR ≥ II), which correlated with higher 1-year mortality (19). In summary, there is growing evidence for the importance of indirect annuloplasty with M-TEER especially in FMR, which is reassured by our group's findings.

### Strengths and limitations

This investigation is a single-center observational study with a medium-size cohort. Only patients with FMR were included and therefore, results cannot be applied to all patients with MR, particularly not to those with DMR. At the same time, this represents an important strength of our study as we investigated only one entity and avoided possible confounding factors related to DMR. The cut-off value for A-Pd reduction that we used in our analysis was calculated based on our patient cohort and may vary in different populations. However, the number of included subjects is large enough to allow statistical evaluation. Moreover, the median of A-Pd reduction in our patient cohort is similar to previous investigations (8, 12). Finally, we did not perform follow-up TEE reevaluating the MV annulus for durability of the observed acute changes which limits long-term interpretation. Especially, volume status may influence annular geometry over time and was not investigated in this study.

In our study A-Pd change ≥6.3% (binary variable) was a significant predictor of the composite endpoint, while % A-Pd change as a continuous variable did not remain a significant predictor. Hence, the use of a binary variable might possibly overestimate the impact of AP diameter change.

Prospective and multicenter studies at best need to further evaluate the role of A-Pd reduction and indirect annuloplasty in M-TEER.

## Conclusion

Our findings indicate that effects of M-TEER in FMR are not limited to the reduction of MR severity, but further entail an impact on annular geometry. Moreover, A-Pd reduction, which mediates indirect annuloplasty, significantly impacts mid-term clinical outcome independent of residual MR. Extensive A-Pd reduction is the prerequisite for annular remodeling in patients with FMR treated with M-TEER. Therefore, periprocedural imaging and assessment should also include annular dimensions and remodeling besides standard evaluation of residual MR. Future longitudinal multicenter studies with larger number of participants and longer follow-up will determine the importance of comprehensive 3D periprocedural assessment of MV annular geometry and its alterations on outcome in patients with different types of MR (FMR vs. DMR).

## Data Availability

The original contributions presented in the study are included in the article/**Supplementary Material**, further inquiries can be directed to the corresponding author.
