# Supplementary material for: Transcatheter edge-to-edge-repair of functional mitral regurgitation induces significant remodeling of mitral annular geometry
Source: Front Cardiovasc Med. 2023 Jun 23;10:1143702. doi: 10.3389/fcvm.2023.1143702 (PMC10326617; doi:10.3389/fcvm.2023.1143702)
Supplement: Supplementary file 1 [file Datasheet1.docx]

| **Supplemental Table 1. 4D MV Analysis grouped according to the Combined Endpoint of Death or Rehospitalization for Heart Failure** | | | | |
| --- | --- | --- | --- | --- |
|  | **Endpoint not reached**  **N=118** | **Endpoint reached**  **N=23** | **Total**  **N=141** | **p-Value** |
| A-Pd pre (cm) | 4.0±0.5 | 4.1±0.6 | 4.0±0.5 | 0.22 |
| A-Pd post (cm) | 3.7±0.6 | 3.9±0.7 | 3.8±0.6 | 0.19 |
| mean relative change (%) | -6.3±10.1 | -5.3±6.5 | -6.15±9.5 | 0.64 |
| **p (pre-post)** | **<0.01** | **<0.01** | **<0.01** |  |
| AL-PMd pre (cm) | 4.1±0.4 | 4.1±0.6 | 4.1±0.5 | 0.91 |
| AL-PMd post (cm) | 4.2±0.5 | 4.4±0.6 | 4.3±0.5 | 0.15 |
| mean relative change (%) | 3.1±8.8 | 7.0±8.7 | 3.73±8.9 | 0.051 |
| **p (pre-post)** | **<0.01** | **<0.01** | **<0.01** |  |
| Nonplanar Angle pre (°) | 152.2±11.3 | 150.5±13.1 | 152.0±11.6 | 0.52 |
| Nonplanar Angle post (°) | 149.1±13.4 | 152.9±14.5 | 149.7±13.6 | 0.51 |
| mean relative change (%) | -1.8±8.9 | 2.1±11.5 | -1.2±9.5 | 0.07 |
| **p (pre-post)** | **0.01** | 0.48 | 0.055 |  |
| AC pre (cm) | 13.4±1.3 | 13.6±1.7 | 13.5±1.4 | 0.57 |
| AC post (cm) | 13.3±1.5 | 13.7±1.8 | 13.3±1.5 | 0.27 |
| mean relative change (%) | -1.2±6.2 | 0.5±7.0 | -0.9±6.4 | 0.24 |
| **p (pre-post)** | **0.03** | 0.84 | 0.061 |  |
| 2D AA pre (cm^2^) | 12.9±2.6 | 13.3±3.5 | 13.0±2.7 | 0.48 |
| 2D AA post (cm^2^) | 12.5±2.8 | 13.6±3.7 | 12.7±3.0 | 0.10 |
| mean relative change (%) | -2.7±12.4 | 3.1±15.4 | -1.8±13.1 | 0.052 |
| **p (pre-post)** | **<0.01** | 0.47 | **0.049** |  |
| 3D AA pre (cm^2^) | 13.5±3.0 | 13.9±3.5 | 13.6±3.1 | 0.64 |
| 3D AA post (cm^2^) | 13.0±2.9 | 14.0±3.7 | 13.13±3.1 | 0.14 |
| mean relative change (%) | -3.6±13.3 | 1.9±15.3 | -2.67±13.7 | 0.08 |
| **p (pre-post)** | **<0.01** | 0.84 | **0.01** |  |
| Tenting Volume pre (cm^3^) | 4.9±2.6 | 4.6±3.0 | 4.8±2.6 | 0.71 |
| Tenting Volume post (cm^3^) | 4.6±2.4 | 5.1±2.9 | 4.7±2.6 | 0.32 |
| mean relative change (%) | -0.4±36.9 | 15.3±36.6 | 2.07±37.2 | 0.07 |
| **p (pre-post)** | 0.07 | 0.13 | 0.27 |  |
| Tenting Area pre (cm2) | 2.6±1.1 | 3.1±1.8 | 2.7±1.2 | 0.052 |
| Tenting Area post (cm2) | 2.3±1.0 | 2.5±1.1 | 2.4±1.0 | 0.43 |
| mean relative change (%) | 0.01±62.2 | -6.1±36.8 | -1.0±58.8 | 0.65 |
| **p (pre-post)** | **<0.01** | 0.11 | **<0.01** |  |
| Annular Height pre (cm) | 0.9±0.2 | 1.6±3.2 | 1.04±1.3 | **0.02** |
| Annular Height post (cm) | 0.9±0.2 | 0.8±0.3 | 0.9±0.3 | 0.29 |
| mean relative change (%) | -2.2±28.7 | -13.9±31.3 | -4.11±29.3 | 0.08 |
| **p (pre-post)** | **0.03** | 0.22 | 0.11 |  |
| Values are shown as mean ± standard deviation (SD); p(pre-post) refers to testing for paired variables.  AA: Annular area, AC: Annular circumference, AL-PMd: anterolateral-posteromedial diameter, A-Pd: Anterior-posterior diameter, MV: mitral valve. | | | | |

| **Supplemental Table 2. Univariate Cox regression for possible predictors of the combined endpoint.** | | | |
| --- | --- | --- | --- |
|  | **HR** | **95% CI** | **p** |
| Age (years) | 1.00 | 0.96-1.05 | 0.91 |
| BMI (kg/m^2^) | 0.99 | 0.92-1.07 | 0.74 |
| Female, N (%) | 1.09 | 0.48-2.47 | 0.84 |
| Arterial Hypertension, N (%) | 1.43 | 0.49-4.21 | 0.51 |
| CAD, N (%) | 0.6 | 0.26-1.35 | 0.21 |
| Prior MI | 1.01 | 0.41-2.44 | 0.99 |
| Hyperlipidemia, N (%) | 0.95 | 0.41-2.19 | 0.95 |
| Pulmonary Hypertension, N (%) | 0.98 | 0.32-2.99 | 0.98 |
| COPD, N (%) | 1.56 | 0.46-5.26 | 0.47 |
| Family Disposition, N (%) | 0.99 | 0.3-3.34 | 0.99 |
| AFib, N (%) | 1.31 | 0.52-3.31 | 0.57 |
| CRT-D/P, N (%) | 0.78 | 0.18-3.33 | 0.74 |
| DCM, N (%) | 1.29 | 0.62-2.69 | 0.49 |
| NYHA class | 2.57 | 1.19-5.54 | **0.02** |
| Troponin T pre (µg/L) | 1.007 | 0.96-1.07 | 0.8 |
| NT-proBNP pre (pg/mL) | 1.07 | 1.02-1.13 | **0.01** |
| eGFR (ml/min) | 0.99 | 0.97-1.01 | 0.28 |
| BB, N (%) | 0.83 | 0.28-2.44 | 0.74 |
| ACEI, N (%) | 0.65 | 0.22-1.92 | 0.44 |
| ARB, N (%) | 0.81 | 0.32-2.05 | 0.65 |
| ARNI, N (%) | 1.96 | 0.86-4.47 | 0.11 |
| MRA, N (%) | 0.82 | 0.36-1.87 | 0.64 |
| SGLT-2 Inhibitors, N (%) | 0.57 | 0.14-2.45 | 0.45 |
| Loop Diuretics, N (%) | 1.34 | 0.4-4.52 | 0.63 |
| Statins, N (%) | 0.69 | 0.3-1.60 | 0.39 |
| ASS, N (%) | 0.56 | 0.19-1.66 | 0.3 |
| NOAC, N (%) | 1.22 | 0.52-2.87 | 0.66 |
| P2Y12 inhibitor, N (%) | 1.4 | 0.33-5.96 | 0.65 |
| LVEF % | 0.98 | 0.95-1.02 | 0.35 |
| LVEDd (mm) | 0.99 | 0.96-1.04 | 0.91 |
| LVEDV (mm) | 0.99 | 0.99-1.003 | 0.39 |
| LVESd (mm) | 1.02 | 0.98-1.05 | 0.35 |
| LVESV (ml) | 0.99 | 0.99-1.004 | 0.34 |
| LA Diameter (mm) | 0.98 | 0.94-1.003 | 0.5 |
| TAPSE (mm) | 0.90 | 0.80-1.02 | 0.1 |
| sPAP (mmHg) | 0.99 | 0.96-1.02 | 0.51 |
| Average Grade of TR | 0.81 | 0.52-1.25 | 0.34 |
| Severe TR, N (%) | 0.88 | 0.35-2.24 | 0.79 |
| Average Grade of MR pre | 2.05 | 0.81-5.19 | 0.13 |
| Average Grade of MR post | 1.33 | 0.62-2.83 | 0.47 |
| Mean PG (mmHg) pre | 1.03 | 0.76-1.39 | 0.86 |
| ERO A (cm^2^) | 3.64 | 0.24-54.15 | 0.35 |
| Vena contracta (mm) | 1.12 | 0.99-1.26 | 0.08 |
| PISA (cm) | 1.24 | 0.13-11.51 | 0.85 |
| MR RV (ml) | 0.99 | 0.98-1.02 | 0.87 |
| Mean PG (mmHg) post | 0.91 | 0.63-1.33 | 0.63 |
| MV orifice area pre (cm^2^) | 1.003 | 0.74-1.36 | 0.99 |
| MV orifice area post (cm^2^) | 1.2 | 0.92-1.55 | 0.18 |
| Number of implanted devices | 0.43 | 0.16-1.16 | 0.1 |
| A-Pd pre (cm) | 1.78 | 0.74-4.31 | 0.2 |
| A-Pd post (cm) | 1.61 | 0.82-1.61 | 0.17 |
| mean relative change (%) | 1.01 | 0.97-1.05 | 0.63 |
| A-Pd reduction ≥6.3% | 0.41 | 0.17-0.98 | **0.046** |
| AL-PMd pre (cm) | 1.04 | 0.42-2.56 | 0.94 |
| AL-PMd post (cm) | 1.8 | 0.81-3.98 | 0.15 |
| mean relative change (%) | 1.04 | 1.001-1.09 | **0.04** |
| Nonplanar Angle pre (°) | 0.99 | 0.95-1.02 | 0.49 |
| Nonplanar Angle post (°) | 1.02 | 0.99-1.05 | 0.22 |
| mean relative change (%) | 1.04 | 0.99-1.08 | 0.06 |
| AC pre (cm) | 1.09 | 0.81-1.49 | 0.57 |
| AC post (cm) | 1.12 | 0.89-1.52 | 0.26 |
| mean relative change (%) | 1.04 | 0.98-1.11 | 0.20 |
| 2D AA pre (cm^2^) | 1.06 | 0.91-1.23 | 0.48 |
| 2D AA post (cm^2^) | 1.12 | 0.98-1.28 | 0.1 |
| mean relative change (%) | 1.03 | 1.002-1.06 | **0.04** |
| 3D AA pre (cm^2^) | 1.03 | 0.90-1.18 | 0.65 |
| 3D AA post (cm^2^) | 1.11 | 0.97-1.26 | 0.13 |
| mean relative change (%) | 1.03 | 1.0-1.06 | 0.06 |
| Tenting Volume pre (cm^3^) | 0.97 | 0.82-1.14 | 0.69 |
| Tenting Volume post (cm^3^) | 1.08 | 0.93-1.27 | 0.31 |
| mean relative change (%) | 1.01 | 1.00-1.02 | 0.06 |
| Tenting Area pre (cm2) | 1.35 | 1.01-1.1 | **0.046** |
| Tenting Area post (cm2) | 1.18 | 0.8-1.75 | 0.41 |
| mean relative change (%) | 1.0 | 0.99-1.01 | 0.67 |
| Annular Height pre (cm) | 1.18 | 1.4-1.35 | **0.01** |
| Annular Height post (cm) | 0.4 | 0.07-2.27 | 0.30 |
| mean relative change (%) | 0.99 | 0.97-1.00 | 0.8 |
| BMI = Body Mass Index (kg/m^2^); CAD = Coronary Artery Disease, COPD = Chronic Obstructive Pulmonary Disease, AF= Atrial Fibrillation, LBBB = Left Bundle Branch Block, CRT = Cardiac Resynchronization Therapy, DCM = Dilatative Cardiomyopathy, NYHA = New York Heart Association, STS = Society of Thoracic Surgeons; NT-proBNP = N-terminal pro hormone brain natriuretic peptide, eGFR = estimated glomerular filtration rate; BP = Blood Pressure, BB = Beta Blocker; ACEI = Angiotensin Converting Enzyme Inhibitor; ARB = AT Receptor Blocker; ARNI = Angiotensin-Neprilysin Inhibitor; MRA = Mineralocorticoid Receptor Antagonist, SGLT-2 = Sodium-Glucose Cotransporter-2; ASS =acetylic salicylic acid ; NOAC= novel oral anticoagulant ; P2Y12 inhibitor = adenosine diphosphate receptor antagonists, MR = mitral regurgitation; MV = mitral valve, LVEF = Left-ventricular Ejection Fraction, LVEDd = Left-ventricular end-diastolic diameter; LVEDV = Left-ventricular end-diastolic Volume ; LVESd= Left-ventricular end-systolic diameter; LVESV = Left-ventricular end-systolic Volume; LA= Left Atrium; IVSd= Septum diameter; TAPSE = Tricuspid Annular Plane Systolic Excursion, sPAP = Systolic Pulmonary Artery Pressure; TR = Tricuspid Regurgitation; PG = pressure gradient; ERO A = effective regurgitant orifice area; PISA = proximal isovelocity surface area; RV = regurgitant volume, AA: Annular area, AC: Annular circumference, AL-PMd: anterolateral-posteromedial diameter, A-Pd: Anterior-posterior diameter, MV: mitral valve. | | | |

| **Supplemental Table 3. 4D MV analysis in atrial vs. ventricular FMR** | | | | |
| --- | --- | --- | --- | --- |
|  | **Total**  **(N=141)** | **atrial FMR**  **(N=24)** | **ventricular FMR**  **(N=117)** | **p-Value** |
| A-Pd pre (cm) | 4.0±0.5 | 4.0±0.5 | 4.0±0.5 | 0.83 |
| A-Pd post (cm) | 3.8±0.6 | 3.7±0.5 | 3.9±0.6 | 0.48 |
| mean relative change (%) | -6.2±9.5 | -8.2±8.6 | -5.7±9.7 | 0.25 |
| **p (pre-post)** | **<0.01** | **<0.01** | **<0.01** |  |
| AL-PMd pre (cm) | 4.1±0.5 | 3.9±0.5 | 4.2±0.4 | **0.01** |
| AL-PMd post (cm) | 4.3±0.5 | 4.1±0.6 | 4.3±0.5 | **0.04** |
| mean relative change (%) | 3.7±8.9 | 4.4±7.7 | 3.6±9.1 | 0.67 |
| **p (pre-post)** | **<0.01** | **0.01** | **<0.01** |  |
| Nonplanar Angle pre (°) | 152.0±11.6 | 152.2±1.0 | 151.9 | 0.93 |
| Nonplanar Angle post (°) | 149.7±13.6 | 149.3±15.3 | 149.8±13.3 | 0.87 |
| mean relative change (%) | -1.2±9.5 | -1.8±9.6 | -1.1±9.5 | 0.74 |
| **p (pre-post)** | 0.055 | 0.32 | 0.1 |  |
| AC pre (cm) | 13.5±1.4 | 13.0±1.6 | 13.5±1.3 | 0.1 |
| AC post (cm) | 13.3±1.5 | 12.9±1.6 | 13.4±1.5 | 0.11 |
| mean relative change (%) | -0.9±6.4 | -1.0±6.8 | -0.9±6.3 | 0.94 |
| **p (pre-post)** | 0.061 | 0.35 | 0.11 |  |
| 2D AA pre (cm^2^) | 13.0±2.7 | 13.0±1.6 | 13.1±2.7 | 0.16 |
| 2D AA post (cm^2^) | 12.7±3.0 | 12.0±3.3 | 12.8±2.9 | 0.25 |
| mean relative change (%) | -1.8±13.1 | -1.0±14.0 | -1.9±12.9 | 0.75 |
| **p (pre-post)** | **0.049** | 0.53 | 0.06 |  |
| 3D AA pre (cm^2^) | 13.6±3.1 | 12.7±3.2 | 13.8±3.0 | 0.13 |
| 3D AA post (cm^2^) | 13.1±3.1 | 12.4±3.3 | 13.3±3.0 | 0.22 |
| mean relative change (%) | -2.7±13.7 | -1.6±13.9 | -2.9±13.7 | 0.68 |
| **p (pre-post)** | 0.26 | 0.35 | **0.02** |  |
| Tenting Volume pre (cm^3^) | 4.8±2.6 | 3.0±2.1 | 5.2±2.6 | **<0.01** |
| Tenting Volume post (cm^3^) | 4.7±2.6 | 3.0±2.1 | 5.0±2.5 | **<0.01** |
| mean relative change (%) | 2.1±37.2 | 0.6±42.2 | 2.4±36.4 | 0.84 |
| **p (pre-post)** | 0.27 | 0.94 | 0.25 |  |
| Tenting Area pre (cm2) | 2.7±1.2 | 1.9±0.9 | 2.8±1.2 | **<0.01** |
| Tenting Area post (cm2) | 2.4±1.1 | 1.6±0.7 | 2.5±1.0 | **<0.01** |
| mean relative change (%) | -1.0±58.8 | -11.6±114.8 | -3.6±38.7 | 0.25 |
| **p (pre-post)** | **<0.01** | 0.12 | 0.01 |  |
| Annular Height pre (cm) | 1.0±1.3 | 0.9±0.2 | 1.1±1.4 | 0.5 |
| Annular Height post (cm) | 0.9±0.3 | 0.8±0.2 | 0.9±0.3 | 0.11 |
| mean relative change (%) | -4.1±29.3 | -6.0±27.3 | -3.7±29.8 | 0.73 |
| **p (pre-post)** | 0.11 | 0.11 | 0.14 |  |
| Values are shown as mean ± standard deviation (SD); p(pre-post) refers to testing for paired variables.  AA: Annular area, AC: Annular circumference, AL-PMd: anterolateral-posteromedial diameter, A-Pd: Anterior-posterior diameter, MV: mitral valve. | | | | |

| **Supplemental Table 4. Comparison of annular geometry in patients with optimal (MR≤I) and non-optimal (MR≥II) results of M-TEER** | | | | |
| --- | --- | --- | --- | --- |
|  | **Total**  **(N=141)** | **Optimal Result**  **(****MR≤I)**  **N=111** | **Non-Optimal Result**  **(MR≥II)**  **N=30** | **p-Value** |
| A-Pd pre (cm) | 4.0±0.5 | 3.9±0.5 | 4.2±0.4 | **0.01** |
| A-Pd post (cm) | 3.8±0.6 | 3.7±0.6 | 4.0±0.6 | **<0.01** |
| mean relative change (%) | -6.2±9.5 | -6.7±9.5 | -4.2±9.7 | 0.2 |
| **p (pre-post)** | **<0.01** | **<0.01** | **0.03** |  |
| AL-PMd pre (cm) | 4.1±0.5 | 4.1±0.4 | 4.3±0.5 | 0.053 |
| AL-PMd post (cm) | 4.3±0.5 | 4.2±0.5 | 4.4±0.5 | 0.06 |
| mean relative change (%) | 3.7±8.9 | 3.7±9.4 | 3.8±6.8 | 0.98 |
| **p (pre-post)** | **<0.01** | **<0.01** | **0.01** |  |
| Nonplanar Angle pre (°) | 152.0±11.6 | 1514±11.6 | 153.9±11.5 | 0.31 |
| Nonplanar Angle post (°) | 149.7±13.6 | 148.7±13.1 | 153.4±14.9 | 0.09 |
| mean relative change (%) | -1.2±9.5 | -1.5±8.7 | 0.2±11.9 | 0.38 |
| **p (pre-post)** | 0.055 | **0.02** | 0.89 |  |
| AC pre (cm) | 13.5±1.4 | 13.3±1.4 | 14.1±1.4 | **0.01** |
| AC post (cm) | 13.3±1.5 | 13.1±1.5 | 14.0±1.4 | **0.01** |
| mean relative change (%) | -0.9±6.4 | -1.1±6.7 | -0.3±4.8 | 0.55 |
| **p (pre-post)** | 0.061 | 0.07 | 0.62 |  |
| 2D AA pre (cm^2^) | 13.0±2.7 | 12.6±2.6 | 14.1±3.0 | **0.01** |
| 2D AA post (cm^2^) | 12.7±3.0 | 12.3±2.9 | 13.4±2.9 | **0.01** |
| mean relative change (%) | -1.8±13.1 | -2.1±13.9 | -0.5±9.7 | 0.57 |
| **p (pre-post)** | **0.049** | 0.06 | 0.54 |  |
| 3D AA pre (cm^2^) | 13.6±3.1 | 13.3±3.0 | 13.7±3.0 | **0.01** |
| 3D AA post (cm^2^) | 13.1±3.1 | 12.8±3.0 | 14.5±2.9 | **0.01** |
| mean relative change (%) | -2.7±13.7 | -3.0±14.6 | -1.3±9.9 | 0.55 |
| **p (pre-post)** | 0.26 | **0.02** | 0.32 |  |
| Tenting Volume pre (cm^3^) | 4.8±2.6 | 4.7±2.5 | 5.4±2.9 | 0.21 |
| Tenting Volume post (cm^3^) | 4.7±2.6 | 4.5±2.5 | 5.1±2.5 | 0.26 |
| mean relative change (%) | 2.1±37.2 | 1.2±37.7 | 5.4±37.7 | 0.59 |
| **p (pre-post)** | 0.27 | 0.41 | 0.42 |  |
| Tenting Area pre (cm2) | 2.7±1.2 | 2.6±1.2 | 3.0±1.4 | 0.15 |
| Tenting Area post (cm2) | 2.4±1.1 | 2.3±1.0 | 2.5±1.0 | 0.37 |
| mean relative change (%) | -1.0±58.8 | -0.01±62.1 | -4.5±45.2 | 0.71 |
| **p (pre-post)** | **<0.01** | **<0.01** | 0.07 |  |
| Annular Height pre (cm) | 1.0±1.3 | 1.1±1.5 | 1.0±0.4 | 0.92 |
| Annular Height post (cm) | 0.9±0.3 | 0.9±0.2 | 0.9±0.3 | 0.61 |
| mean relative change (%) | -4.1±29.3 | -3.4±29.5 | -6.4±29.1 | 0.6 |
| **p (pre-post)** | 0.11 | 0.17 | 0.11 |  |
| Values are shown as mean ± standard deviation (SD); p(pre-post) refers to testing for paired variables.  AA: Annular area, AC: Annular circumference, AL-PMd: anterolateral-posteromedial diameter, A-Pd: Anterior-posterior diameter, MV: mitral valve. | | | | |

| **Supplemental Table 5. Comparison of annular geometry in patients with decreasing and increasing A-Pd** | | | | |
| --- | --- | --- | --- | --- |
|  | **Total**  **(N=141)** | **% (-)A-Pd**  **(N=116)** | **% (+) A-Pd**  **(N=25)** | **p-Value** |
| Average grade of MR pre | 3.6±0.5 | 3.6±0.5 | 3.5±0.5 | 0.2 |
| MR Grade III, N (%) | 57 (40.4) | 44 (37.9) | 13 (52.0) | 0.19 |
| MR Grade IV, N (%) | 84 (59.6) | 72 (62.1) | 12 (48.0) |  |
| Average Grade of MR post | 1.1±0.6 | 1.1±0.6 | 1.3±0.5 | 0.1 |
| MR Grade ≤I | 111 (78.7) | 93 (80.2) | 18 (72.0) | 0.37 |
| MR Grade II | 30 (21.2) | 23 (19.8) | 7 (28.0) |  |
| Mean PG (mmHg) pre | 2.3±1.5 | 2.1±1.1 | 3.2±2.3 | **<0.01** |
| ERO A (cm^2^) | 0.3±0.1 | 0.3±0.1 | 0.3±0.2 | 0.97 |
| Vena contracta (mm) | 8.8±3.0 | 8.9±3.0 | 8.0±2.7 | 0.31 |
| PISA (cm) | 0.8±0.2 | 0.8±0.2 | 0.7±0.2 | 0.71 |
| MR RV (ml) | 39.2±19.6 | 40.1±20.7 | 34.7±19.1 | 0.26 |
| Mean PG (mmHg) post | 3.1±1.2 | 2.9±1.2 | 3.8±1.2 | **0.02** |
| MV orifice area pre (cm^2^) | 4.1±1.5 | 4.1±1.6 | 3.6±0.9 | 0.24 |
| MV orifice area post (cm^2^) | 2.7±1.5 | 2.8±1.5 | 2.5±2.0 | 0.5 |
| Number of implanted devices | 1.4±0.5 | 1.4±0.5 | 1.4±0.5 | 0.52 |
| Device type |  |  |  |  |
| NT&NTR&NTW | 43 (30.5) | 37 (31.9) | 6 (24.0) | **0.03** |
| XT&XTR&XTW | 25 (17.7) | 23 (19.8) | 2 (8.0) |  |
| PASCAL P10 | 33 (23.4) | 29 (25.0) | 4 (16.0) |  |
| PASCAL Ace | 40 (28.4) | 27 (23.3) | 13 (52.0) |  |
| A-Pd pre (cm) | 4.0±0.5 | 4.0±0.5 | 3.9±0.5 | 0.32 |
| A-Pd post (cm) | 3.8±0.6 | 3.7±0.5 | 4.2±0.6 | **<0.01** |
| mean relative change (%) | -6.2±9.5 | -9.3±6.8 | 8.1±7.2 | **<0.01** |
| **p (pre-post)** | **<0.01** | **<0.01** | **<0.01** |  |
| AL-PMd pre (cm) | 4.1±0.5 | 4.1±0.5 | 4.0±0.5 | 0.27 |
| AL-PMd post (cm) | 4.3±0.5 | 4.3±0.5 | 4.2±0.5 | 0.68 |
| mean relative change (%) | 3.7±8.9 | 3.3±7.6 | 5.7±13.4 | 0.23 |
| **p (pre-post)** | **<0.01** | **<0.01** | 0.08 |  |
| Nonplanar Angle pre (°) | 152.0±11.6 | 151.8±11.4 | 152.6±11.4 | 0.75 |
| Nonplanar Angle post (°) | 149.7±13.6 | 150.2±14.1 | 147.5±11.0 | 0.38 |
| mean relative change (%) | -1.2±9.5 | -0.8±9.9 | -3.0±6.9 | 0.28 |
| **p (pre-post)** | 0.055 | 0.22 | **0.03** |  |
| AC pre (cm) | 13.5±1.4 | 13.5±1.4 | 13.3±1.3 | 0.41 |
| AC post (cm) | 13.3±1.5 | 13.2±1.5 | 13.8±1.5 | 0.063 |
| mean relative change (%) | -0.9±6.4 | -2.1±5.3 | 4.7±7.7 | **<0.01** |
| **p (pre-post)** | 0.061 | **<0.01** | **0.01** |  |
| 2D AA pre (cm^2^) | 13.0±2.7 | 12.5±2.6 | 12.5±2.8 | 0.32 |
| 2D AA post (cm^2^) | 12.7±3.0 | 12.5±3.0 | 13.6±2.8 | 0.1 |
| mean relative change (%) | -1.8±13.1 | -4.4±10.4 | 10.0±16.9 | **<0.01** |
| **p (pre-post)** | **0.049** | **<0.01** | **0.01** |  |
| 3D AA pre (cm^2^) | 13.6±3.1 | 13.7±3.1 | 13.0±3.1 | 0.28 |
| 3D AA post (cm^2^) | 13.1±3.1 | 12.5±3.0 | 14.2±3.0 | 0.06 |
| mean relative change (%) | -2.7±13.7 | -5.4±11.2 | 10.2±16.8 | **<0.01** |
| **p (pre-post)** | 0.26 | **<0.01** | **0.01** |  |
| Tenting Volume pre (cm^3^) | 4.8±2.6 | 4.8±2.6 | 4.8±2.6 | 0.9 |
| Tenting Volume post (cm^3^) | 4.7±2.6 | 4.5±2.5 | 5.2±2.5 | 0.27 |
| mean relative change (%) | 2.1±37.2 | -1.4±35.5 | 17.4±41.4 | **0.02** |
| **p (pre-post)** | 0.27 | 0.07 | 0.25 |  |
| Tenting Area pre (cm2) | 2.7±1.2 | 2.7±1.2 | 2.6±1.1 | 0.71 |
| Tenting Area post (cm2) | 2.4±1.1 | 2.3±1.0 | 2.7±1.1 | **0.048** |
| mean relative change (%) | -1.0±58.8 | -4.3±60.7 | 14.3±47.1 | 0.15 |
| **p (pre-post)** | **<0.01** | **<0.01** | 0.44 |  |
| Annular Height pre (cm) | 1.0±1.3 | 1.1±1.5 | 0.9±0.3 | 0.69 |
| Annular Height post (cm) | 0.9±0.3 | 0.8±0.2 | 1.0±0.2 | **0.032** |
| mean relative change (%) | -4.1±29.3 | -6.0±30.5 | 4.8±21.1 | 0.09 |
| **p (pre-post)** | 0.11 | 0.1 | 0.72 |  |
| Values are shown as mean ± standard deviation (SD); p(pre-post) refers to testing for paired variables.  MR = mitral regurgitation; MV = mitral valve, PG = pressure gradient; ERO A = effective regurgitant orifice area; PISA = proximal isovelocity surface area; RV = regurgitant volume, AA: Annular area, AC: Annular circumference, AL-PMd: anterolateral-posteromedial diameter, A-Pd: Anterior-posterior diameter, MV: mitral valve. | | | | |


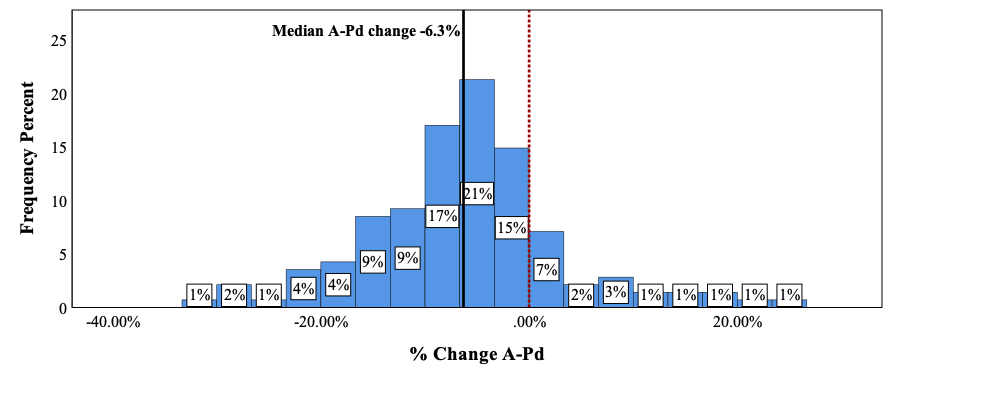


Supplemental Figure 1: Histogram showing distribution of relative (%) A-Pd change. The Median of A-Pd change is indicated with a black line. The dashed red line divides patients with decreasing A-Pd from those with increasing A-Pd.
